# Supplementary material for: The development of adolescents’ loneliness during the COVID-19 pandemic: The role of peer status and contact with friends
Source: PLoS One. 2023 May 26;18(5):e0286085. doi: 10.1371/journal.pone.0286085 (PMC10218743; doi:10.1371/journal.pone.0286085)
Supplement: S1 Appendix — (DOCX) [file pone.0286085.s001.docx]

# S1 Appendix. Latent class analysis and multigroup latent growth curve analysis peer status groups

## Latent class analysis

Table A1 provides the results of the latent class analysis (LCA) that provides peer status groups. The entropy was excellent for all group-solutions. The aBIC value was lower in the third group than in the second group and further decreased after adding the fifth group. The LMR-LRT showed that adding a fourth group did not significantly improve the model fit compared to the 3-group solution, indicating the 3-group solution as the best fit. Given these fit indices, we selected the 3-group solution.

**Table S1A Latent Class Analysis Peer Status Groups N=512**

| Solution | aBIC | Entropy | LMR-LRT | Group Prevalence (%) | | | | |
| --- | --- | --- | --- | --- | --- | --- | --- | --- |
|  |  |  |  | 1 | 2 | 3 | 4 | 5 |
| 2-group | -2130.127 | .996 | *p* < .001 | 95.1% | 4.9% |  |  |  |
| **3-group** | **-2322.616** | **.929** | ***p* = .04** | **84%** | **11.3%** | **4.7%** |  |  |
| 4-group | -2833.181 | .944 | *p* = .15 | 71.3% | 13% | 12.5% | 3.3% |  |
| 5-group | -3022.595 | .951 | *p* = .29 | 71.3% | 13% | 12.5% | 1.2% | 2.2% |

*Note.* aBIC = sample-size adjusted Bayesian Information Criterion. LMR-LRT = Lo-Mendell-Rubin Likelihood Ratio Test.

## Multigroup latent growth curve analysis

Table A2 shows the fit indices for the fully constrained and the unconstrained models. The unconstrained multigroup model fitted better than the constrained multigroup model (Δχ^2^(12) = 61.76, *p* < .001, ΔCFI = .254). More stringent testing revealed that the unconstrained model fitted significantly better in Δχ^2^ compared with partly constrained models in which intercepts or slopes were held equal for different peer status groups. Therefore, the unconstrained model was used as the final model. The final model had acceptable to good model fit (χ^2^(10) = 13.22, *p* = .212, RMSEA = .043, SRMR = .035, CFI = .982).

**Table S1B Model Fit Indices of the Model Comparisons N=512**

| Parameter | χ^2^ | df | *p* | RMSEA | SRMR | CFI | Δχ^2^ | Δdf | *p* | ΔCFI |
| --- | --- | --- | --- | --- | --- | --- | --- | --- | --- | --- |
| Fully constrained | 68.46 | 18 | <.001 | .128 | .128 | .721 |  |  |  |  |
| Unconstrained | 13.22 | 10 | .212 | .043 | .035 | .982 | 58.50 | 8 | <.001 | .261 |

*Note.* RMSEA = Root Means Square Error of Approximation. SRMR = Standardized Root Mean Squared Residual. CFI = Comparative Fit Index. Slope variances of the *normative* and *rejected* were constrained to 0 because of negative residual variance.
