# Supplementary material for: The development of adolescents’ loneliness during the COVID-19 pandemic: The role of peer status and contact with friends
Source: PLoS One. 2023 May 26;18(5):e0286085. doi: 10.1371/journal.pone.0286085 (PMC10218743; doi:10.1371/journal.pone.0286085)
Supplement: S2 Appendix — (DOCX) [file pone.0286085.s002.docx]

# S2 Appendix. Latent class analysis and multigroup latent growth curve analysis contact profiles

## Latent class analysis

Table B1 shows the results of the exploratory latent class analysis on type and amount of contact during the school shutdown. The aBIC value was the lowest in the 5-class solution. The entropy was good for all class-solutions. The LMR-LRT showed that adding a fourth class did not significantly improve the model fit compared with the 3-class solution. Therefore, the 3-class solution was selected.

**Table S2A Latent Class Analysis Contact Profiles N=505**

| Solution | aBIC | Entropy | LMR-LRT | Group Prevalence (%) | | | | |
| --- | --- | --- | --- | --- | --- | --- | --- | --- |
|  |  |  |  | 1 | 2 | 3 | 4 | 5 |
| 2-class | 4211.77 | .939 | *p* < .001 | 24.2% | 75.8% |  |  |  |
| **3-class** | **4054.76** | **.945** | ***p* < .001** | **6.1%** | **19.8%** | **74.1%** |  |  |
| 4-class | 3940.12 | .958 | *p* = .869 | 19.8% | 5.2% | 20.6% | 54.5% |  |
| 5-class | 3050.10 | .999 | *p* = .500 | 3.6% | 3.0% | 30.9% | 62.6% | 0.0% |

*Note.* aBIC = sample-size adjusted Bayesian Information Criterion. LMR-LRT = Lo-Mendell-Rubin Likelihood Ratio Test.

## Multigroup latent growth curve analysis

Table B2 shows the model fit indices for the fully constrained and the unconstrained multigroup model, gender included as unconstrained in both models. The unconstrained multigroup model fitted better than the constrained multigroup model (Δχ2(8) = 33.11, *p* < .001, ΔCFI = .064). More stringent testing revealed improved model fit when intercepts of loneliness were held equal for the *low contact* profile and the *high all-round contact* profile and that the slopes of loneliness could be held equal for the *high contact but low (video)calling* profile and the *high contact* profile. However, reflecting on the theorized differences between the *low contact* and the *high all-round contact* profile, holding intercepts equal for these profiles cannot be justified. A partly constrained model in which only the slopes of loneliness development were held equal for the *high contact but low (video)calling* profile and the *high all-round contact* profile did not significantly differ in model fit from the unconstrained model (Δχ^2^(1) = 0.15, *p* = .696). Therefore, the unconstrained model was used as the final model. The unconstrained model had a mediocre to acceptable model fit (χ^2^(10) = 41.76, *p* < .001, RMSEA = 0.137, SRMR = 0.058, CFI = 0.919).

Modification indices in M*plus* suggested to include the correlation between the residuals of loneliness in Jan/Feb ‘20 and March-May ‘20 for the first (*low contact*) and third (*high all-round contact*) profile. Adding this to the model led to a better fit, particularly in RMSEA which improved from 0.137 to 0.071, without changing the results and conclusions from the analysis. Including the correlation between the residuals of loneliness for the measure in March-May ‘20 and Oct/Nov ‘20 while constraining the correlations between the residuals per time point, and adding this for the second (*high contact but low (video)calling*) profile was theoretically necessary. This only led to a negligible improvement in model fit, with the RMSEA improving from .137 to .131. Therefore, we used the most parsimonious model without correcting for the correlation between the residuals of the repeated measures of loneliness.

**Table S2B Model Fit Indices of the Model Comparisons N=512**

| Parameter | χ^2^ | df | *p* | RMSEA | SRMR | CFI | Δχ^2^ | Δdf | *p* | ΔCFI |
| --- | --- | --- | --- | --- | --- | --- | --- | --- | --- | --- |
| Fully constrained | 74.67 | 18 | <.001 | .137 | .093 | .855 |  |  |  |  |
| Unconstrained | 41.76 | 10 | <.001 | .137 | .058 | .919 | 33.11 | 8 | <.001 | .064 |

*Note.* RMSEA = Root Means Square Error of Approximation. SRMR = Standardized Root Mean Squared Residual. CFI = Comparative Fit Index. Slope variances of the *low* contact and *high all-round* contact model were constrained to 0 because of negative residual variance.
