# Supplementary material for: The development of adolescents’ loneliness during the COVID-19 pandemic: The role of peer status and contact with friends
Source: PLoS One. 2023 May 26;18(5):e0286085. doi: 10.1371/journal.pone.0286085 (PMC10218743; doi:10.1371/journal.pone.0286085)
Supplement: Attachment — Submitted filename: Response to Reviewers.docx [file pone.0286085.s003.docx]

Rebuttal PLOS ONE

Manuscript ‘The Development of Adolescents’ Loneliness during the COVID-19 Pandemic: The Role of Peer Status and Contacts with Friends’

| **Journal Requirements** | |
| --- | --- |
| 1. Please ensure that your manuscript meets PLOS ONE's style requirements, including those for file naming. | Done. |
| 2. Please provide additional details regarding participant consent. In the ethics statement in the Methods and online submission information, please ensure that you have specified (1) whether consent was informed and (2) what type you obtained (for instance, written or verbal, and if verbal, how it was documented and witnessed). If your study included minors, state whether you obtained consent from parents or guardians. If the need for consent was waived by the ethics committee, please include this information. | Done. |
| 3. In your Data Availability statement, you have not specified where the minimal data set underlying the results described in your manuscript can be found. PLOS defines a study's minimal data set as the underlying data used to reach the conclusions drawn in the manuscript and any additional data required to replicate the reported study findings in their entirety. All PLOS journals require that the minimal data set be made fully available. For more information about our data policy, please see <http://journals.plos.org/plosone/s/data-availability>.  "Upon re-submitting your revised manuscript, please upload your study’s minimal underlying data set as either Supporting Information files or to a stable, public repository and include the relevant URLs, DOIs, or accession numbers within your revised cover letter. For a list of acceptable repositories, please see <http://journals.plos.org/plosone/s/data-availability#loc-recommended-repositories>. Any potentially identifying patient information must be fully anonymized.  Important: If there are ethical or legal restrictions to sharing your data publicly, please explain these restrictions in detail. Please see our guidelines for more information on what we consider unacceptable restrictions to publicly sharing data: <http://journals.plos.org/plosone/s/data-availability#loc-unacceptable-data-access-restrictions>. Note that it is not acceptable for the authors to be the sole named individuals responsible for ensuring data access.  We will update your Data Availability statement to reflect the information you provide in your cover letter. | See the cover letter for our updated Data Availability statement. |
| 4. Please ensure that you include a title page within your main document. You should list all authors and all affiliations as per our author instructions and clearly indicate the corresponding author. | Done. In addition, we made our preregistration public and disclosed the name of the project from which the data was used. |
| 5. Please amend your manuscript to include your abstract after the title page. | Done. |
| 6. Please include your full ethics statement in the ‘Methods’ section of your manuscript file. In your statement, please include the full name of the IRB or ethics committee who approved or waived your study, as well as whether or not you obtained informed written or verbal consent. If consent was waived for your study, please include this information in your statement as well. | Done. |
| 7. Please include captions for your Supporting Information files at the end of your manuscript, and update any in-text citations to match accordingly. Please see our Supporting Information guidelines for more information: <http://journals.plos.org/plosone/s/supporting-information>. | Done. |

| **Additional Editor Comments** | |
| --- | --- |
| 1. As you can see from the reviewers' comments, some changes are suggested, which I agree with. | We thank you for the comprehensive review of our manuscript and the opportunity to revise and resubmit our manuscript. We considered and in almost all cases followed. |
| 2. In addition, I stress the importance of reviewing the text with a native English speaker. | The text is now corrected by a native English speaker. |

| **Reviewer #1** | |
| --- | --- |
| 1. Does this online survey conform to the recommended standards for conducting and reporting web-based surveys, i.e., the Checklist for Reporting Results of Internet E-surveys (CHERRIES)? I suggest specifying this point. | The CHERRIES does not apply. The PRIMS project did not use online web- or email surveys. We selected schools based on a stratified sample design with the sample frame consisting of the full population of Dutch primary schools, resulting in a representative sample on the school level. We describe this under methods. We added a reference to the PRIMS data collection report and codebook (Zwier et al., 2023) for more information on the sampling, testing of surveys, and data collection. |
| 2.  I recommend further improving the references about the impact of the COVID-19 pandemic on adolescents worldwide by citing some of these recent studies on the topic: DOI: 10.3389/fpsyg.2020.559951; DOI: 10.1016/j.jad.2021.04.016; DOI: 10.1089/cyber.2020.0478. | We included the third reference in our Discussion under ‘The role of contact with friends’ on p. 33. In addition, we included other references to relevant recent studies such as Repo et al., 2022, Espinoza & Hernandez, 2022 and Lampraki et al., 2022.  We included neither the first mentioned reference (because it did not measure loneliness) nor the second reference (because loneliness was a predictor rather than the outcome of interest). |
| 3. It would be interesting to compare the experience of adolescents with that of university students to highlight similarities and differences. In this regard, it may be helpful for authors to read some recently published articles on this topic: DOI: 10.1016/j.psychres.2020.113111; DOI: 10.13129/2282-1619/mjcp-3009; DOI: 10.1038/s41598-022-21288-z. | We agree that this comparison is interesting and added it to the Discussion under ‘Average loneliness development’ on p. 31. In that discussion we decided to refer to longitudinal studies on loneliness. |

| **Reviewer #2** | |
| --- | --- |
| 1. Please revise the English. Even if the manuscript is overall easy to follow and understand, there are some areas where the authors could polish the phrasing. For example, at line 6 to 7 "These societal concerns were of special concern during the COVID-19 pandemic" I would encourage the authors to find a synonym for "concern", perhaps. This is just an example, please revise the whole manuscript. Furthermore, I would encourage the authors to revise the introduction section. I would welcome a somewhat more robust argumentation of the importance of this study. Also, it could be helpful if the overall introduction was revised - the arguments provided by the authors seem somewhat fragmented, I would want to see more coherence and flow. | The text is now reviewed by a native English speaker. We think this improved the coherence and flow of the text. Concerning the introduction: we have completely rewritten the first paragraph of the introduction to address this. The importance of the study is now central. |
| 2. If you choose to provide graphics, please make sure that they are easy to read. The ones provided are very blurry and hard to follow. Also, I would replace figure 3 and 4 with tables containing percentages/means. This is somewhat a personal preference, as I find more easy to follow a table than a graph. If the authors choose to retain the graphs, please add the percentages/means to each graph. | We replaced figures 3 and 4 with tables. We saved the other figures in high resolution as .tiff files so they are not blurry. |
| 3. I would welcome a more robust introduction with a clearer emphasis on the importance of the study. I would also try to be more concise, as at times I had the impression of reading sections of the procedure/measurement. | We have rewritten the first paragraph of the introduction to emphasize the importance of the study. We dropped methodological language in the introduction. |
| 4. The discussion section conveys the personal interpretation of the authors, however, I would like to see a more theoretically based interpretation of the findings. Furthermore, I would welcome further practical implications of the findings. | We removed the personal interpretation of declining loneliness for victimized students from the discussion and expanded upon this in the practical implications. We included more references in the discussion and removed subjective wording. Furthermore, we expanded upon our practical implications. |
| 5. In the Results - Descriptive statistics section, there are some things I would welcome more details about. For example, the authors mention "Stability in students’ feelings of loneliness was moderate to strong". What does this mean? I see a correlation afterwards and I imagine that it is the correlation for LACA scores from T1 correlated to T2 and T2 with T3 (measures)? Also I am puzzled by the choice of words, stability. It is highly expected that the scores from the same instrument correlate.. Maybe there is a nuance there that the authors could shed some light on. | We indeed refer to the moderate to strong correlations, giving information on how the LACA scores are correlated over time. We understand that the choice of words for ‘stability’ is puzzling. We changed this by speaking of how the loneliness measures ‘relate’ over time when discussing the correlations. |
| 6. In the Results - Descriptive statistics section, the authors say "Although measured in the same survey, feelings of loneliness differed between March-May ‘20 and Oct/Nov ‘20", and then provide a correlation score. In the light of the previous understanding of the statistics linked to the stability, I have to admit that this is confusing. Furthermore, when differences are mentioned, I usually expect a T test, not necessarily a correlation. Could the authors indicate the reasoning/rephrase this section? | In Oct/Nov ’20 students filled in their loneliness at that moment and retrospectively their loneliness during the lockdown. The correlation between their two loneliness scores is moderate, which suggests that the students realized that the loneliness measures referred to different periods. We understand our wording was confusing as we aim to address the validity of the measures rather than provide the descriptives. For that reason, we moved this explanation to the measures paragraph (under ‘loneliness’) rather than explaining it as descriptives. We changed the wording ‘differed’ to ‘not strongly related’ to indicate we used a correlation. |
